# Mechanical power normalized to lung-thorax compliance indicates weaning readiness in prolonged ventilated patients

**DOI:** 10.1038/s41598-021-03960-y

**Published:** 2022-01-07

**Authors:** Alessandro Ghiani, Joanna Paderewska, Swenja Walcher, Konstantinos Tsitouras, Claus Neurohr, Nikolaus Kneidinger

**Affiliations:** 1 Lung Center Stuttgart – Schillerhoehe Lung Clinic (Affiliated to the Robert-Bosch-Hospital GmbH, Stuttgart), Department of Pulmonology and Respiratory Medicine, Solitudestrasse 18, 70839 Gerlingen, Germany; 2 Department of Internal Medicine V, Ludwig-Maximilians-University (LMU) of Munich, Munich, Germany; 3 Comprehensive Pneumology Center (CPC-M), Member of the German Center for Lung Research (DZL), Munich, Germany

**Keywords:** Medical research, Risk factors

## Abstract

Since critical respiratory muscle workload is a significant determinant of weaning failure, applied mechanical power (MP) during artificial ventilation may serve for readiness testing before proceeding on a spontaneous breathing trial (SBT). Secondary analysis of a prospective, observational study in 130 prolonged ventilated, tracheotomized patients. Calculated MP’s predictive SBT outcome performance was determined using the area under receiver operating characteristic curve (AUROC), measures derived from k-fold cross-validation (likelihood ratios, Matthew's correlation coefficient [MCC]), and a multivariable binary logistic regression model. Thirty (23.1%) patients failed the SBT, with absolute MP presenting poor discriminatory ability (MCC 0.26; AUROC 0.68, 95%CI [0.59‒0.75], *p* = 0.002), considerably improved when normalized to lung-thorax compliance (LTC_dyn_-MP, MCC 0.37; AUROC 0.76, 95%CI [0.68‒0.83], *p* < 0.001) and mechanical ventilation P_a_CO_2_ (so-called power index of the respiratory system [PI_rs_]: MCC 0.42; AUROC 0.81 [0.73‒0.87], *p* < 0.001). In the logistic regression analysis, PI_rs_ (OR 1.48 per 1000 cmH_2_O^2^/min, 95%CI [1.24‒1.76], *p* < 0.001) and its components LTC_dyn_-MP (1.25 per 1000 cmH_2_O^2^/min, [1.06‒1.46], *p* < 0.001) and mechanical ventilation P_a_CO_2_ (1.17 [1.06‒1.28], *p* < 0.001) were independently related to SBT failure. MP normalized to respiratory system compliance may help identify prolonged mechanically ventilated patients ready for spontaneous breathing.

## Introduction

Mechanical ventilation, the core characteristic of intensive care, is a life-saving procedure for patients presenting severe respiratory failure. However, since prolonged ventilation is associated with increased morbidity and mortality^1,2^, liberation from the ventilator should begin as early as possible. A two-step strategy towards extubation, involving readiness testing and subsequent spontaneous breathing trials (SBT), has been shown to reduce the length of ventilation and costs of medical care in the ICU^3^.

So far, apart from clinical factors (e. g., evidence of clinical improvement, P/F ratio, adequate hemodynamics), several prediction variables (e.g., maximum inspiratory pressure, tidal volume, or minute ventilation) have been assessed in a one-time measurement for readiness testing^4–6^, with most of these traditional weaning predictors used to distinguish between patients who can or cannot sustain a trial of spontaneous breathing usually determined after a short period of self-ventilation following disconnection from the ventilator^4^. Serial measurement of esophageal pressure at the beginning of the SBT, reflecting patients’ respiratory effort and breathing work, has improved further diagnostic accuracy in predicting weaning failure^7^, most probably because it reflects the underlying pathophysiology, namely an imbalance between respiratory impedance and neuromuscular capacity^8,9^. However, esophageal pressure measurement for assessment of patients’ work of breathing, due to its complexity, is rarely used in daily routine. Moreover, not total power output (pressure times volume per time unit) but critical stress imposed on respiratory muscles (e. g. expressed as pressure–time product or tension-time index of the diaphragm) seems to be the major determinant of weaning failure^8,10^.

Mechanical power (MP) of artificial ventilation, the energy transferred to the respiratory system per time unit, is a chief determinant of adequate gas exchange. Basically, the MP concept was introduced to shed further light on the underlying mechanisms behind ventilator-induced lung injury, as it unifies all ventilatory variables deemed responsible^11^. However, MP may also indicate respiratory muscle workload during spontaneous breathing, contributing significantly to weaning failure^7–9^. Accordingly, in a retrospective analysis, mechanical power normalized to lung-thorax compliance, a surrogate of applied power per unit of ventilated lung volume (consistent with stress intensity), was independently associated with the outcome of a spontaneous breathing trial^12^.

In this analysis, we evaluated the discriminatory performance of mechanical power in predicting the outcome of a short weaning trial and thus indicate weaning readiness.

## Methods

Secondary analysis of a previously reported prospective observational cohort study conducted at a specialized weaning center that focused on predictors of prolonged ventilation weaning failure^13^. The study was performed following the Declaration of Helsinki and approved by the local institutional review board for human studies (Ethics Committee of the State Chamber of Physicians of Baden-Wuerttemberg, Germany, file number F-2018-116). All patients or a legal representative gave their written informed consent to participate. We now report MP as a candidate predictor of the first spontaneous breathing trial upon patient admission to our center.

### Patient selection

**Figure 1:**
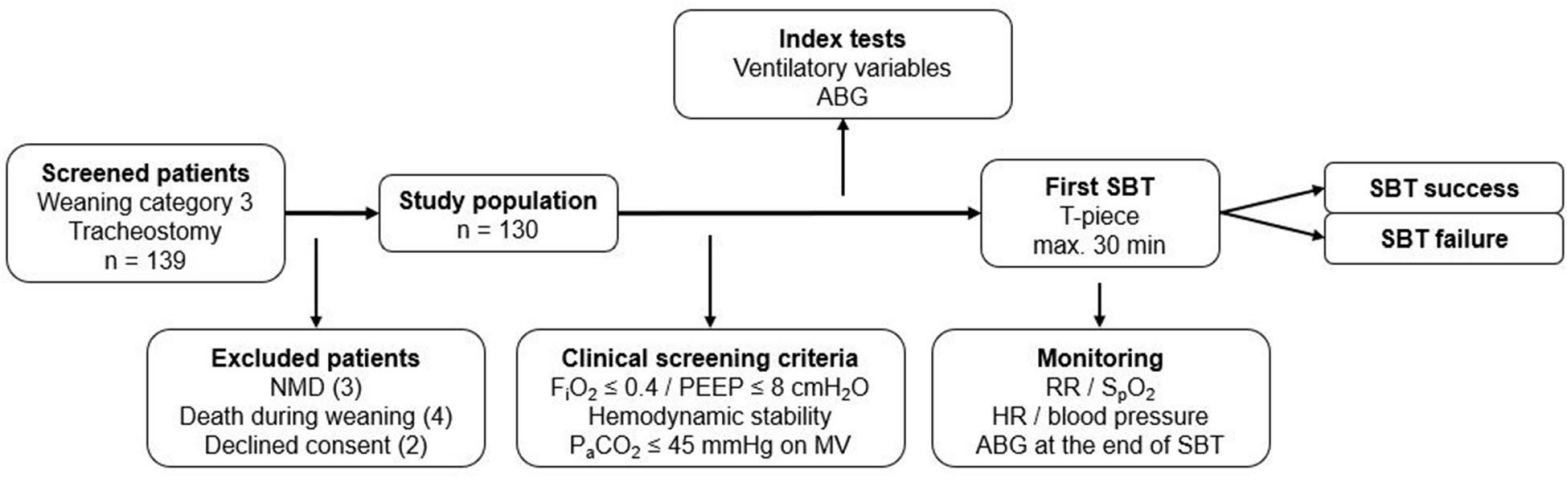
Study flow. Abbreviations: NMD, neuromuscular disease; F_i_O_2_, fractional inspired oxygen; PEEP, positive end-expiratory pressure; MV, mechanical ventilation; ABG, arterial blood gas analysis; MP, mechanical power; SBT, spontaneous breathing trial; RR, respiratory rate; HR, heart rate.

Only tracheotomized patients referred for prolonged weaning, classified as Category 3 defined by Boles and co-workers^14^, were analyzed. Patients were excluded from the study if they had a diagnosis of neuromuscular disease, died before weaning completion, or declined consent (Fig. 1).

### Spontaneous breathing trials

All patients were ventilated in the pressure-controlled, assist-control (A/C) mode (Vivo 50 & 60, Breas Medical AB, Moelnlycke, Sweden). The main characteristic of this ventilation mode is that the same (high) pressure is applied during both an assisted and controlled breath, consistent with full ventilator support intending to defend alveolar ventilation and minimizing respiratory muscle activity, thereby recovering from the imposed work of breathing between SBT.

The first SBT upon admission to the weaning center occurred as soon as pre-defined clinical screening criteria for weaning readiness were met, which were (1) adequate oxygenation (S_p_O_2_ ≥ 92%) with fractional inspired oxygen ≤ 0.40 and PEEP ≤ 8 cmH_2_O, (2) hemodynamic stability without the need for vasopressors or inotropic agents, and (3) normocapnia (P_a_CO_2_ ≤ 45.0 mmHg) on mechanical ventilation. Patients were not sedated when they underwent the SBT.

In general, the first SBT occurred in the early morning. Patients were placed in the semi-recumbent position for these trials. A few minutes before SBT started, ventilatory variables were obtained from the ventilators’ display in a one-time measurement, and an arterial blood gas analysis (ABG) was performed simultaneously. The patient was then disconnected from the ventilator and breathed room air for 30 min through a T-piece with oxygen admixture at the same level as during mechanical ventilation. The first SBT was always performed under the supervision of a respiratory therapist, and vital signs were continuously monitored to detect respiratory distress immediately. Another ABG was performed at the end of SBT.

### Candidate predictors of weaning

The following parameters were calculated from ventilatory variables and the corresponding arterial blood gas values collected immediately before the first SBT (Fig. 1): ∆P_aw_ (dynamic driving pressure, defined as P_max_—PEEP in the pressure-controlled ventilation mode), dynamic lung-thorax compliance (LTC_dyn_)^15^, mechanical power^11^ using the simplified formula proposed by Becher and colleagues^16,17^, and ventilatory ratio (VR, a surrogate for dead space ventilation)^18^.

Total MP was further normalized to (1) the predicted body weight (a surrogate of the total lung capacity of a healthy individual, PBW-MP)^19^ and (2) LTC_dyn_ (indicating actual ventilated lung volume, LTC_dyn_-MP)^12^. To allow comparability between individual respiratory rate and ventilator pressure settings, LTC_dyn_-MP was ultimately corrected for corresponding mechanical ventilation P_a_CO_2_ (simulating isocapnic conditions), called the Power index of the respiratory system^13^. Further details can be found in the Supplementary information.

### Classification of outcome

Failure of the SBT was defined as the occurrence of objective clinical signs of respiratory failure (breathing frequency > 35/min, tachycardia > 130 bpm, systolic blood pressure > 160 mmHg, or S_p_O_2_ < 88% despite increasing oxygen admixture) and/or changes in blood gas values consistent with ventilatory failure (hypercapnia [P_a_CO_2_ > 45.0 mmHg] with or without respiratory acidosis [pH < 7.35])^14^.

### Statistical analysis

Descriptive and frequency statistics were used to summarize patients’ demographics and baseline characteristics. As appropriate, differences between groups in categorical variables were analyzed using Chi-square or Fisher's exact tests. Continuous variables were subjected to the Kolmogorov–Smirnov normality test for homogeneity of variance. Depending on the statistical distribution, either Student's *t*-test or Mann–Whitney *U*-test was used to examine differences in these parameters.

Discriminatory weaning outcome ability of the selected variables was assessed through a receiver operating characteristic (ROC) curve analysis with diagnostic accuracy expressed as area under the ROC curve (AUROC). Moreover, we conducted a prospective, stratified, 2-times repeated, 2-fold cross-validation^20^ (Supplementary Fig. S1, Fig. S2, and Table S1), with the predictive performance of each index expressed as sensitivity, specificity, positive/negative predictive value, accuracy, positive/negative likelihood ratio, diagnostic odds ratio (DOR), F_1_ score, and Matthew's correlation coefficient (MCC).

We also performed a binary logistic regression analysis to derive variables independently associated with SBT failure from baseline clinical characteristics, comorbidities, and the candidate predictors computed from the ventilatory variables. Variables considered a priori clinically relevant, and variables with a *p* value less than 0.2 in the bivariate analysis were included in the multivariable models (using forward selection). The model's goodness-of-fit was evaluated with the Hosmer & Lemeshow test and Nagelkerke R^2^. We reported odds ratios (OR) with 95% confidence intervals (95%CI).

Finally, Pearson's *r* was used to correlate the index tests with SBT duration and P_a_CO_2_ at the end of the weaning trial.

All tests were two-tailed; statistical significance was indicated by *p* < 0.05. All analyses were performed using MedCalc statistical software version 19.2.5 (MedCalc Software Ltd, Ostend, Belgium). Further details on the statistical methods can be found in the supplementary information.

### Ethics approval and consent to participate

The study was approved by the local institutional review board for human studies (Ethics Committee of the State Chamber of Physicians of Baden-Wuerttemberg, Germany, file number F-2018-116) and performed following the Declaration of Helsinki. Written informed consent was obtained from all patients or a legal representative.

### Consent for publication

Not applicable.

## Results

One-hundred and thirty-nine consecutive patients were screened between March 2019 and August 2020, of whom 130 (93.5%) were included in the study. Nine patients were excluded from the analyses; three had a diagnosis of neuromuscular disease, four died before weaning completion, and two declined to participate.

Clinical characteristics differed between patients with successful and failed SBT regarding smoking history, presence of chronic obstructive pulmonary disease (COPD) and malignancy, and acute exacerbation of COPD as the primary reason for intubation with mechanical ventilation (Table 1).

**Table 1:** Clinical characteristics on admission to the weaning center—comparison of patients with successful and unsuccessful first SBT.

Clinical characteristics	All patients (n = 130)	SBT success (n = 100)	SBT failure (n = 30)	*p* value^a^
Age (years)	69 (60–76)	69 (59–76)	70 (65–72)	0.923^c^
Gender (male)	82 (63.1)	66 (66.0)	16 (53.3)	0.209^d^
Body mass index (kg/m^2^)	26.1 (23.0–31.1)	25.5 (22.9–30.9)	28.3 (24.2–33.3)	0.085^c^
Obesity (BMI ≥ 30 kg/m^2^)	40 (30.8)	28 (28.0)	12 (40.0)	0.213^d^
Smoking history	48 (36.9)	29 (29.0)	19 (63.3)	**<** **0.001**^**d**^
APACHE-II (points)	16 (12–19)	16 (12–19)	16 (12–20)	0.772^b^
Albumin (g/dL)	2.1 (1.8–2.5)	2.1 (1.8–2.5)	2.1 (1.9–2.6)	0.269^c^
Ventilator days on admission	25 (16–34)	25 (16–33)	27 (16–38)	0.333^c^
Intubation to tracheostomy (days)	12 (7–18)	12 (7–18)	10 (6–16)	0.445^c^
ECLA	14 (10.8)	13 (13.0)	1 (3.3)	0.136^d^
**Reason for mechanical ventilation**				
Pneumonia	51 (39.2)	37 (37.0)	14 (46.7)	0.343^d^
Surgery	32 (24.6)	28 (28.0)	4 (13.3)	0.103^d^
Cardiopulmonary resuscitation	10 (7.7)	7 (7.0)	3 (10.0)	0.590^d^
Acute exacerbation of COPD	10 (7.7)	4 (4.0)	6 (20.0)	**0.004**^**d**^
Acute heart failure	6 (4.6)	5 (5.0)	1 (3.3)	1.000^e^
Sepsis (including septic shock)	7 (5.4)	6 (6.0)	1 (3.3)	1.000^e^
Other	17 (13.1)	13 (13.0)	4 (13.3)	0.962^d^
**Comorbidities**				
Charlson comorbidity index (points)	5 (4–7)	5 (4–7)	6 (4–7)	0.507^c^
Renal insufficiency	46 (35.4)	36 (36.0)	10 (33.3)	0.789^d^
Hemodialysis	24 (18.5)	19 (19.0)	5 (16.7)	0.678^d^
Diabetes mellitus	35 (26.9)	25 (25.0)	10 (33.3)	0.369^d^
Coronary artery disease	33 (25.4)	25 (25.0)	8 (26.7)	0.854^d^
COPD	30 (23.1)	16 (16.0)	14 (46.7)	**<** **0.001**^**d**^
Chronic heart failure	17 (13.1)	15 (15.0)	2 (6.7)	0.234^d^
Malignancy	10 (7.7)	5 (5.0)	5 (16.7)	**0.036**^**d**^
Hepatopathy	7 (5.4)	6 (6.0)	1 (3.3)	1.000^e^
Interstitial lung disease	8 (6.2)	7 (7.0)	1 (3.3)	0.681^e^

Continuous variables are presented as median (– interquartile range [IQR]; categorical variables are presented as number (%).

BMI, body mass index; APACHE-II, Acute Physiology and Chronic Health Evaluation II score; ECLA, extracorporeal lung assistance (during acute respiratory failure); COPD, chronic obstructive pulmonary disease.

^a^*p* value for differences between patients with successful and unsuccessful SBT.

^b^Student's *t*-test.

^c^Mann-Whitney *U*-test.

^d^Chi-squared test.

^e^Fisher's exact test.

Significant values are in Bold.

Failure of the SBT occurred in 30 patients (23.1%) (Supplementary Table S2), with significant differences observed in ventilatory variables and MP indices (Table 2).

**Table 2:** Results of first SBT—comparison of patients with successful and unsuccessful SBT.

First SBT	All patients (n = 130)	SBT success (n = 100)	SBT failure (n = 30)	*p* value^a^
Time from admission to first SBT (days)	1 (0–3)	1 (0–3)	1 (1–3)	0.638^b^
Hemoglobin on first SBT (g/dL)	8.3 (± 1.2)	8.3 (± 1.2)	8.1 (± 1.3)	0.266^c^
**Ventilatory variables & MP indices**				
F_i_O_2_	0.27 (± 0.05)	0.27 (± 0.05)	0.28 (± 0.04)	0.156^c^
Tidal volume (mL)	547 (± 86)	547 (± 83)	547 (± 97)	0.459^c^
Minute ventilation (L/min)	9.0 (± 1.9)	9.0 (± 2.0)	9.0 (± 1.7)	0.676^c^
P_a_CO_2_ on MV (mmHg)	35.0 (± 5.4)	33.9 (± 4.8)	38.8 (± 4.5)	**<** **0.001**^**b**^
Ventilatory ratio	1.19 (± 0.34)	1.14 (± 0.34)	1.38 (± 0.32)	**<** **0.001**^**c**^
PEEP (cmH_2_O)	6.0 (± 0.9)	6.0 (± 0.9)	6.0 (± 0.8)	0.717^c^
P_max_ (cmH_2_O)	23.6 (± 4.2)	22.8 (± 4.1)	26.3 (± 3.5)	**<** **0.001**^**b**^
∆P_aw_ (cmH_2_O)	17.6 (± 4.0)	16.8 (± 3.9)	20.3 (± 3.4)	**<** **0.001**^**c**^
LTC_dyn_ (mL/cmH_2_O)	32.8 (± 9.6)	34.3 (± 9.6)	27.9 (± 7.9)	**<** **0.001**^**c**^
Mechanical power (J/min)	21.0 (± 5.9)	20.2 (± 5.7)	23.6 (± 5.8)	**0.004**^**c**^
PBW-MP (J/min/kg)	0.3295 (± 0.1106)	0.3111 (± 0.1033)	0.3907 (± 0.1137)	**<** **0.001**^**c**^
LTC_dyn_-MP (cmH_2_O^2^/min)	7167 (± 3063)	6569 (± 2885)	9159 (± 2824)	**<** **0.001**^**c**^
Power index_rs_^1.0^ (cmH_2_O^2^/min)	5696 (± 2884)	5012 (± 2490)	7978 (± 2971)	**<** **0.001**^**c**^
Power index_rs_^2.0^ (cmH_2_O^2^/min)	4636 (± 2896)	3902 (± 2323)	7082 (± 3292)	**<** **0.001**^**c**^
**P**_**a**_**CO**_**2**_ **on ABG**				
P_a_CO_2_ on MV pre-SBT (mmHg)	35.0 (± 5.4)	33.9 (± 4.8)	38.8 (± 5.5)	**0.001**^**b**^
P_a_CO_2_ on SB post-SBT (mmHg)*	38.7 (± 7.0)	36.3 (± 4.6)	48.9 (± 6.5)	**<** **0.001**^**b**^
∆P_a_CO_2_ (post-/pre-SBT)	3.9 (± 5.8)	2.4 (± 4.4)	10.2 (± 7.2)	**<** **0.001**^**c**^
pH on ABG				
pH on MV pre-SBT	7.50 (± 0.05)	7.50 (± 0.05)	7.48 (± 0.05)	0.192^c^
pH on SB post-SBT*	7.46 (± 0.06)	7.48 (± 0.04)	7.40 (± 0.07)	**<** **0.001**^**c**^
∆pH (post-/pre-SBT)	− 0.03 (± 0.06)	− 0.02 (± 0.05)	− 0.09 (± 0.07)	**<** **0.001**^**c**^
**P**_**a**_**O**_**2**_ **on ABG**				
P_a_O_2_ on MV pre-SBT (mmHg)	83.7 (± 15.9)	85.0 (± 15.9)	79.4 (± 15.5)	0.069^c^
P_a_O_2_ on SB post-SBT (mmHg)*	76.9 (± 18.1)	77.4 (± 19.0)	74.7 (± 13.8)	0.790^c^
∆P_a_O_2_ (post-/pre-SBT)	− 7.4 (± 21.0)	− 7.6 (± 22.2)	− 6.4 (± 15.0)	0.506^c^
**Duration of first SBT (min)**	30 (30–30)	30 (30–30)	30 (11–30)	**<** **0.001**^**c**^

Continuous variables are presented as arithmetic means values (± standard deviation) or median (– interquartile range [IQR]); categorical variables are presented as numbers (%).

SBT, spontaneous breathing trial; MP, mechanical power; F_i_O_2_, the fraction of inspired oxygen; MV, mechanical ventilation; PEEP, positive end-expiratory pressure; P_max_, the pre-set inspiratory positive airway pressure; ∆P_aw_, dynamic driving pressure (defined as P_max_—PEEP in the pressure-controlled ventilation mode); LTC_dyn_, dynamic lung-thorax compliance; PBW-MP, mechanical power normalized to predicted body weight; LTC_dyn_-MP, mechanical power normalized to dynamic lung-thorax compliance, ABG, arterial blood gas analysis.

*ABG at the end of the SBT was missing in seven patients due to severe respiratory distress, requiring immediate resumption of mechanical ventilation.

^a^*p* value for differences between patients with successful and unsuccessful SBT.

^b^Student’s *t*-test.

^c^Mann-Whitney *U*-test.

^d^Chi-squared test.

Significant values are in Bold.

Patients' baseline clinical characteristics and outcomes were equally distributed after each randomization for cross-validation (Supplementary Table S1). Supplementary Table S3 shows the mean threshold values derived from the training sets that best predicted SBT failure.

**Figure 2:**
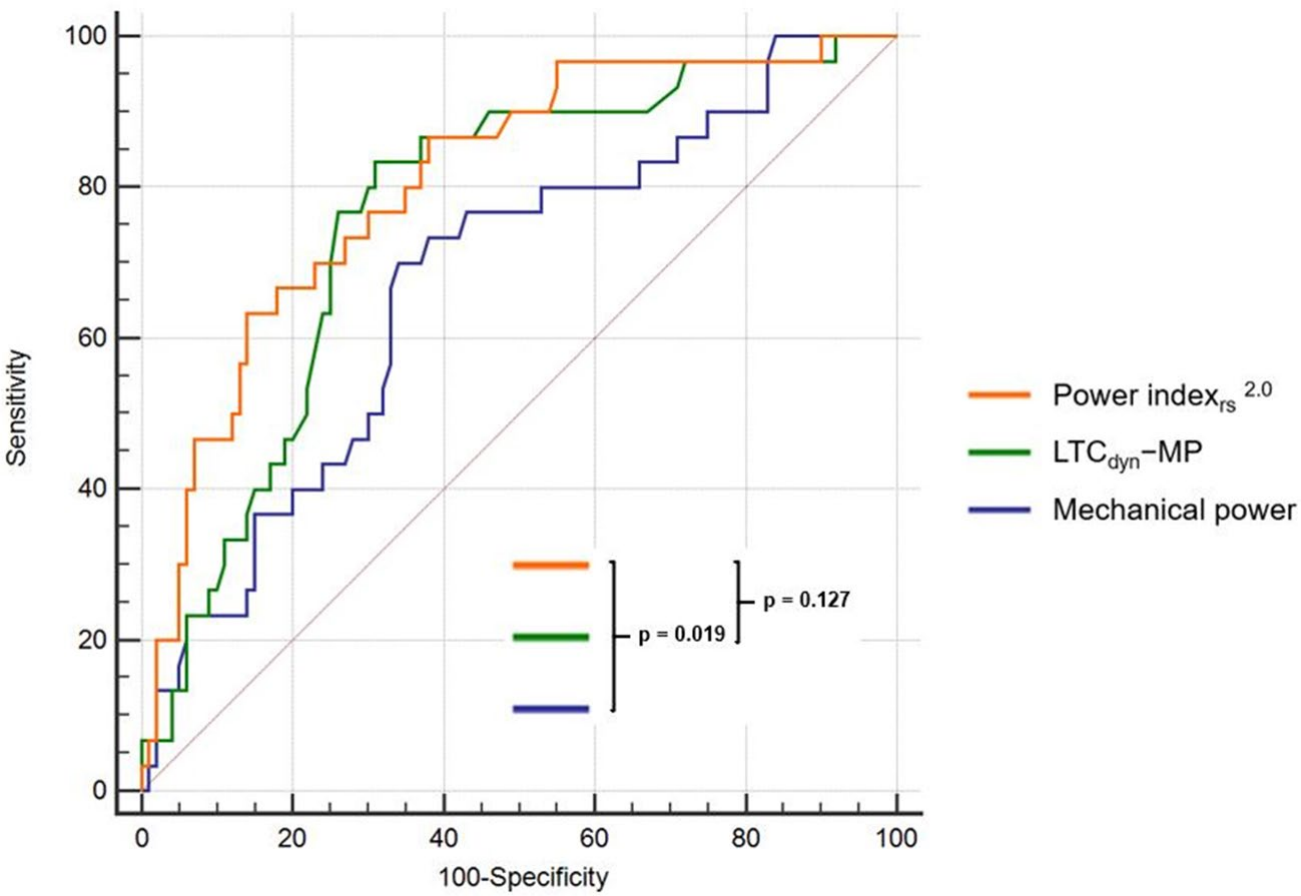
Comparison of ROC curves for mechanical power, LTC_dyn_-MP, and the power index_rs_^2.0^ predicting the outcome of SBT – all patients. Abbreviations: ROC, receiver operating characteristic curve; LTC_dyn_-MP, mechanical power normalized to dynamic lung-thorax compliance.

Absolute MP showed poor diagnostic accuracy (MCC 0.26; AUROC 0.68, 95%CI [0.59‒0.75], *p* = 0.002), but there was a notable increase in discriminatory performance when MP was normalized to surrogates of lung volume (LTC_dyn_-MP: DOR 10.0, MCC 0.37, AUROC 0.76 [95%CI 0.68‒0.83], *p* < 0.001; PBW-MP: DOR 5.3, MCC 0.29, AUROC 0.71 [0.62‒0.79], *p* < 0.001), further improved by correction for corresponding mechanical ventilation P_a_CO_2_ (Power index_rs_: DOR 8.6, MCC 0.42, AUROC 0.81 [0.73‒0.87], *p* < 0.001) (Tables 3, 4, Fig. 2). Most of these indices predominantly showed a higher negative predictive value regarding failure of the SBT.

**Table 3:** Cross-validated performance of variables analyzed to predict the outcome of SBT—mean values derived from the test sets.

Variables	Failure of spontaneous breathing trial									
	Sens	Spec	PPV	NPV	Accuracy	PLR	NLR	DOR	F_1_	MCC
P_a_CO_2_ on MV	68 (40–89)	69 (54–81)	41 (28–54)	88 (77–94)	68 (56–79)	2.4 (1.3–4.2)	0.5 (1.0–0.2)	5.6	0.51	0.32
Ventilatory ratio	68 (41–88)	70 (55–83)	40 (28–54)	88 (78–94)	69 (62–80)	2.3 (1.3–4.0)	0.4 (1.0–0.2)	6.9	0.50	0.33
LTC_dyn_	63 (37–84)	69 (55–81)	37 (25–52)	87 (77–93)	68 (55–79)	2.0 (1.2–3.6)	0.5 (1.0–0.3)	5.1	0.46	0.28
Mechanical power	70 (44–88)	61 (46–74)	34 (24–46)	88 (76–94)	63 (50–74)	1.7 (1.1–2.8)	0.5 (1.1–0.2)	6.7	0.45	0.26
PBW-MP	65 (39–85)	67 (53–79)	39 (26–53)	87 (76–93)	67 (54–78)	2.3 (1.2–4.6)	0.5 (1.0–0.3)	5.3	0.47	0.29
LTC_dyn_-MP	72 (45–89)	71 (57–83)	43 (30–56)	90 (79–95)	71 (59–82)	2.5 (1.2–4.4)	0.4 (0.9–0.2)	10.0	0.53	0.37
Power index_rs_^1.0^	78 (47–93)	72 (58–84)	45 (32–58)	91 (80–96)	72 (60–83)	2.7 (1.6–4.8)	0.3 (0.8–0.1)	8.6	0.57	0.42
Power index_rs_^2.0^	70 (42–90)	75 (60–86)	46 (32–60)	89 (79–95)	73 (61–84)	2.9 (1.6–5.3)	0.4 (0.9–0.2)	7.1	0.55	0.39

Assessment of mean sensitivity and specificity, positive and negative predictive value, positive and negative likelihood ratio, diagnostic odds ratio, F_1_ score, and Matthews correlation coefficient (with 95% confidence intervals).

SBT, spontaneous breathing trial; Sens, sensitivity; Spec, specificity; PPV, positive predictive value; NPV, negative predictive value; PLR, positive likelihood ratio; NLR, negative likelihood ratio; DOR, diagnostic odds ratio; F_1_, F_1_ score; MCC, Matthews correlation coefficient; MV, mechanical ventilation; LTC_dyn_, dynamic lung-thorax compliance; PBW-MP, mechanical power normalized to predicted body weight; LTC_dyn_-MP, mechanical power normalized to dynamic lung-thorax compliance.

**Table 4:** The area under the ROC curve for each variable analyzed to predict the outcome of first SBT—all patients.

Variables	AUROC
P_a_CO_2_ on MV	0.74 (0.66–0.81)
Ventilatory ratio	0.74 (0.65–0.81)
LTC_dyn_	0.71 (0.62–0.78)
Mechanical power	0.68 (0.59–0.75)
PBW-MP	0.71 (0.62–0.79)
LTC_dyn_-MP	0.76 (0.68–0.83)
Power index_rs_^1.0^	0.81 (0.73–0.87)
Power index_rs_^2.0^	0.81 (0.73–0.87)

The accuracy of each variable in the whole study population presented as the area under the ROC curve with 95% confidence intervals.

SBT, spontaneous breathing trial; MV, mechanical ventilation; LTC_dyn_, dynamic lung-thorax compliance; PBW-MP, mechanical power normalized to predicted body weight; LTC_dyn_-MP, mechanical power normalized to dynamic lung-thorax compliance.

In the binary logistic regression analysis, the Power index (OR 1.48 per 1000 cmH_2_O^2^/min, 95%CI [1.24‒1.76], *p* < 0.001) was independently associated with failure of the SBT in univariable, and its components LTC_dyn_-MP (1.25 per 1000 cmH_2_O^2^/min, [1.06‒1.46], *p* < 0.001) and mechanical ventilation P_a_CO_2_ (1.17 [1.06‒1.28], *p* < 0.001) in the multivariable model (R^2^ 0.293) (Table 5, Supplementary Table S4–S6).

**Table 5:** Variables associated with failure of SBT—Results of binary logistic regression analysis.

Variables	Univariable analysis		Multivariable model 1		Multivariable model 2		Multivariable model 3	
	OR (95%CI)	*P*	OR (95%CI)	*P*	OR (95%CI)	*P*	OR (95%CI)	*P*
Age	1.00 (0.97‒1.03)	0.978						
Gender (male)	0.59 (0.26‒1.35)	0.210						
BMI	1.04 (0.98‒1.10)	0.166						
APACHE-II	1.01 (0.94‒1.09)	0.770						
CCI	1.07 (0.89‒1.28)	0.472						
COPD	4.5 (1.88‒11.24)	**<** **0.01**						
Obesity	1.71 (0.73‒4.01)	0.214						
Immunosuppression	0.51 (0.14‒1.85)	0.304						
Malignancy	3.80 (1.02‒14.2)	**0.047**						
VD on admission	1.01 (0.99‒1.03)	0.174						
ECLA	0.23 (0.03‒1.84)	0.166						
Hb on SBT	0.99 (0.95‒1.02)	0.460						
P_a_CO_2_ on MV	1.21 (1.10‒1.33)	**<** **0.01**	1.19 (1.09‒1.31)	**<** **0.01**	1.30 (1.14‒1.47)	**<** **0.01**	1.17 (1.06‒1.28)	**<** **0.01**
Ventilatory ratio	7.47 (1.88‒29.6)	**<** **0.01**			0.08 (0.01‒0.92)	**0.043**		
LTC_dyn_	0.91 (0.86‒0.97)	**<** **0.01**						
Mechanical power	1.10 (1.03‒1.19)	**<** **0.01**	1.09 (1.00‒1.18)	**0.043**				
PBW-MP, per 10^–2^ J/min/kg	1.06 (1.03‒1.11)	**<** **0.01**			1.13 (1.05‒1.21)	**<** **0.01**		
LTC_dyn_-MP, per 1000 cmH_2_O^2^/min	1.32 (1.14‒1.53)	**<** **0.01**					1.25 (1.06‒1.46)	**<** **0.01**
Power index_rs_^1.0^, per 1000 cmH_2_O^2^/min	1.45 (1.22‒1.72)	**<** **0.01**						
Power index_rs_^2.0^, per 1000 cmH_2_O^2^/min	1.48 (1.24‒1.76)	**<** **0.01**						

Multivariable regression models included age, gender, BMI, COPD, malignancy, ventilator days on admission, ECLA, ventilatory ratio, and indices derived from calculated mechanical power.

BMI, body mass index; APACHE-II, Acute Physiology and Chronic Health Evaluation II score; CCI, Charlson comorbidity index; COPD, chronic obstructive pulmonary disease; VD, ventilator days; ECLA, extracorporeal lung assistance; Hb, hemoglobin; SBT, spontaneous breathing trial; MV, mechanical ventilation; LTC_dyn_, dynamic lung-thorax compliance; PBW-MP, mechanical power normalized to predicted body weight; LTC_dyn_-MP, mechanical power normalized to dynamic lung-thorax compliance.

Significant values are in Bold.

Finally, the Power index^2.0^ significantly correlated with the duration of SBT (*r* = − 0.38 [− 0.52 to − 0.22; *p* < 0.001]) (Supplementary Table S7) and with P_a_CO_2_ at end of the weaning trial (*r* = 0.57 [0.43‒0.67]; *p* < 0.001) (Supplementary Fig. S3).

## Discussion

The present analysis aimed to investigate the discriminatory performance of the mechanical power in predicting the outcome of a short weaning trial in prolonged ventilated patients. In summary, absolute MP performed poorly, with improvement by correction for surrogates of lung volume (e. g. LTC_dyn_, so-called specific MP, consistent with stress intensity) and mechanical ventilation P_a_CO_2_, the former allowing comparability between differences in gender and body height, the latter simulating isocapnic conditions by accounting for individual respiratory rate and ventilator pressure settings. Moreover, in the multivariable logistic regression analysis, these indices were independently associated with failure of the SBT.

This approach of combining mechanical ventilation stress intensity with a marker of alveolar ventilation is a simplified method for estimating associated mechanical power (stress intensity times LTC_dyn_) required to defend alveolar ventilation and has recently been shown to correlate well with patients’ ability to wean from prolonged ventilation successfully^13^. For instance, the difference between actual LTC_dyn_-MP and Power index approximates the required change in ventilator stress intensity output (determined by the inspiratory pressures, PEEP, and respiratory rate) necessary to reach the target P_a_CO_2_, which was arbitrary set at 45 mmHg in the present study. As a prerequisite for this, we aimed at near totally unloading the respiratory pump by applying the same (high) inspiratory pressure during both controlled and assisted ventilation (in A/C mode), thereby minimizing patients’ respiratory muscle activity between SBT. In other words, even if patients’ respiratory muscles were relaxed, the applied backup respiratory rate and inspiratory pressure (both of which determine minute ventilation) were sufficient to defend alveolar ventilation. Nevertheless, our technique is a trade-off between the accuracy of MP measurement and its clinical applicability. This approach to weaning is contrary to the current practice of liberation attempts from short-term mechanical ventilation, which is a gradual reduction in ventilator support as soon as weaning readiness is suspected from clinical screening criteria (markers of respiratory system recovery), with an optional assessment of weaning predictors during a short period of spontaneous breathing, followed by a weaning trial^14^. Even so, our approach has been shown to speed up weaning in prolonged ventilated, tracheotomized patients compared to a gradual reduction of pressure support^21^.

Because no single parameter provides a perfect prediction for readiness testing, the combination of variables that indicate when to undertake or refrain from making the first weaning attempt is appealing^4,7,22,23^. Yet, most of the described integrative weaning indices (e.g., CROP, Integrative Weaning Index, or CORE) showed only moderate accuracy in predicting the outcome of a short weaning trial, and none of them were validated in prolonged ventilated patients. Moreover, since most of the included variables were measured during spontaneous breathing, assessment of these indices is more complex, reducing their applicability in daily routine. In contrast, PI_rs_ can be assessed at the bedside without disconnecting the patient from the ventilator.

Weaning predictors are used as a decision point to determine whether a patient may advance to a spontaneous breathing trial. This approach has two main goals. First, it may help identify patients who are not yet ready for a weaning trial, assuming a failed SBT results in worse outcomes. However, in contrast to extubation failure, which is associated with increased mortality^24^, there is so far no evidence that a failed weaning trial could be harmful, provided ventilation is resumed immediately in the event of respiratory distress. In a physiologic study, Laghi and co-workers demonstrated that patients who failed a weaning trial did not develop contractile fatigue of the diaphragm (consistent with muscle injury), potentially leading to subsequent unsuccessful weaning attempts or unsuccessful liberation from the ventilator^25^. Moreover, these investigators were able to show that diaphragmatic recruitment at the end of a failed weaning trial, estimated by the electrical activity of the diaphragm, was half of the maximum, corresponding to reflex inhibition of the diaphragm with at the same time redistribution of motoneuron output to rib cage and accessory muscles, a potential mechanism for the prevention of diaphragmatic contractile fatigue^26^. Accordingly, patients categorized as difficult to wean, failing up to three SBT before successful discontinuation of mechanical ventilation, showed no increase in ICU‒ or hospital mortality compared to patients successfully extubated on the first attempt^27,28^. The second and presumably more important goal of using weaning predictors is to avoid unnecessarily prolonged ventilation, which associates with significant morbidity and mortality^1,2^. In this context, given its higher negative predictive value, the Power index may be particularly useful in identifying patients who are likely to succeed in a trial of spontaneous breathing rather than indicating weaning failure. This finding is in line with most classic prediction variables, which display high sensitivity for weaning success (e. g., the rapid shallow breathing index or maximum inspiratory pressure), but usually lack sufficient specificity^4^. This phenomenon may, in part, be explained by the duration of weaning trials in different studies. Since the relationship between critical stress imposed on respiratory muscles and time to task failure in healthy subjects follows an inverse power function^29^, 30 min of spontaneous breathing may be too short for some patients for the development of apparent respiratory distress or alveolar hypoventilation, heightened muscle effort is not sustained for a sufficient time^25^. Yet, these patients may experience weaning failure in a more extended trial^25^. Accordingly, in the present study, the Power index was significantly correlated with the duration of SBT. Nevertheless, the selected threshold value finally determines LTC_dyn_-MP’s predictive weaning outcome ability, allowing further adjustments depending on the primary goal of using it as a weaning predictor (e.g., preferring a higher sensitivity or specificity for SBT failure).

In a recent observational study, patients determined to have premature or delayed weaning trials, compared to patients with an opportune SBT, showed worse clinical outcomes^30^. However, so far, controlled studies have failed to prove advantages of using weaning predictors (e. g., shortening the duration of mechanical ventilation) compared to decision making based on clinical screening criteria alone^31^. In two randomized trials, incorporating the rapid shallow breathing index (frequency-tidal volume ratio) in a weaning protocol, which was then used (intervention) or not used (control) for the decision to proceed on spontaneous breathing, this approach significantly prolonged weaning time, had no perceptible effect on mortality or extubation failure rates^32^, and thus may not be helpful in a weaning protocol^33^. Consequently, incorporating weaning predictors in the decision to proceed on a weaning trial generally has been questioned^34^, and routine use of such prediction variables is currently not recommended^14,35^.

Weaning and extubation outcomes in intubated patients are two independent processes, the inability to breathe without ventilator assistance due to an imbalance between load and capacity of respiratory muscles (referred to as non-airway failure or weaning failure) and the inability to maintain a patent airway (so-called airway failure)^36^, both of which are associated with different risk factors^37^. This is a significant problem when assessing prediction variables in such patients, limiting the development of a single prediction model with high diagnostic accuracy. Tracheostomy protects the airway during both mechanical and self-ventilation, allowing the evaluation of spontaneous breathing in isolation. Hence, investigating such patients and comparing those who can or cannot defend alveolar ventilation may provide a deeper understanding of why weaning failure occurs.

Our study has several limitations. First, since it was a monocentric analysis, generalizability to other centers (external validity) is uncertain. Second, the first weaning attempt was made over 30 min, which may have been too short, and a more extended trial may have disclosed more patients with weaning failure, affecting diagnostic accuracy of the prediction variables. Finally, since we evaluated only patients with tracheotomies, we cannot extrapolate our results to intubated patients.

## Conclusions

Mechanical power normalized to lung-thorax compliance independently predicted the outcome of a short weaning trial and thus may serve for readiness testing before proceeding on spontaneous breathing in prolonged ventilated patients. Given its higher negative predictive value, this index may be particularly useful in identifying patients ready to attempt spontaneous breathing, thereby probably decreasing ventilation time.

## Supplementary Information

Supplementary Information.

## Data Availability

The datasets used and analyzed during the current study are available from the corresponding author on reasonable request.
