# Supplementary material for: Mechanical power normalized to lung-thorax compliance indicates weaning readiness in prolonged ventilated patients
Source: Sci Rep. 2022 Jan 7;12:6. doi: 10.1038/s41598-021-03960-y (PMC8741981; doi:10.1038/s41598-021-03960-y)
Supplement: Supplementary file 1 — Supplementary Information. [file 41598_2021_3960_MOESM1_ESM.pdf]

Mechanical power normalized to lung-thorax compliance indicates weaning readiness in prolonged ventilated patients

Alessandro Ghiani, MD; Joanna Paderewska, MD; Swenja Walcher, RT;

Konstantinos Tsitouras, MD; Claus Neurohr, MD, and Nikolaus Kneidinger, MD

Supplementary information

1. **Definitions of ventilator variables and mechanical power indices (Page 2)**
2. **Statistical analysis – Metrics of diagnostic accuracy (Page 4)**
3. **Figure S1 (Page 5)**
4. **Figure S2 (Page 6)**
5. **Table S1 (Page 7)**
6. **Table S2 (Page 8)**
7. **Table S3 (Page 8)**
8. **Table S4 (Page 9)**
9. **Table S5 (Page 9)**
10. **Table S6 (Page 10)**
11. **Table S7 (Page 10)**
12. **Figure S3 (Page 11)**
13. **References (Page 12)**

Definitions of ventilator variables and mechanical power indices

**Ventilatory ratio (VR)**	Ventilatory ratio (VR) is a surrogate of pulmonary dead space fraction and a simple bedside index of impaired efficiency of ventilation[1-2]:<br><br>$$\mathbf{VR = VE_{measured} * P_aCO_{2\text{-}measured} / VE_{predicted} * P_aCO_{2\text{-}ideal}}$$<br><br>$VE_{measured}$ is the measured minute ventilation (mL/min), $P_aCO_{2\text{-}measured}$ is the measured arterial pressure of carbon dioxide (mmHg), $VE_{predicted}$ is the predicted minute ventilation calculated as predicted bodyweight x 1000 (mL/min), and P_aCO2_{ideal} is the expected arterial pressure of carbon dioxide in normal lungs if ventilated with the predicted minute ventilation. $P_aCO_{2\text{-}ideal}$ is set at 37.5 mmHg (5 kPa) for all patients. VR is a unitless ratio, and a value approximating one would represent normal ventilating lungs.
**PEEP**	Positive end-expiratory pressure (PEEP)
**P_{max}**	The pre-set inspiratory positive airway pressure (P_{max}), including PEEP
**Dynamic driving pressure (ΔP_{aw})**	Dynamic driving pressure (ΔP_{aw}) in the pressure-controlled ventilation mode was calculated using P_{max} and PEEP:<br><br>$$\mathbf{\Delta P_{aw}\ (cmH_2O) = P_{max} - PEEP}$$
**Dynamic lung-thorax compliance (LTC_{dyn})**	Dynamic lung-thorax compliance (LTC_{dyn}) was calculated using tidal volume (VT) and dynamic driving pressure:<br><br>$$\mathbf{LTC_{dyn}\ (mL/cmH_2O) = VT / \Delta P_{aw}}$$
**Mechanical power (MP)**	Mechanical power (MP)[3] provided by the ventilator in the pressure-controlled mode was calculated using the simplified formula proposed by Becher et al., including VT, respiratory rate (RR), and P_{max}[4-5]:<br><br>$$\mathbf{MP\ (J/min) = 0.098 * VT * RR * P_{max}}$$<br>$$\mathbf{MP\ (J/min) = 0.098 * VE * P_{max}}$$<br><br>With each breath delivered by the ventilator, a certain amount of energy (Joule) is transferred to the patients` respiratory system. This energy is mainly used to overcome the airways' resistance, inflate the lungs, and expand the thoracic cage.
**Mechanical power normalized to predicted body weight (PBW-MP)**	MP normalized to predicted body weight (PBW-MP) provided by the ventilator was calculated using MP and predicted body weight (PBW):<br><br>**PBW-MP (J/min/kg) = MP / PBW**<br><br>Calculation of PBW[6]:<br><br>**PBW (males) = 50 + 0.91 * (body height [cm]–152.4)**<br>**PBW (females) = 45.5 + 0.91 * (body height [cm]–152.4)**

**Mechanical power normalized to dynamic lung-thorax compliance (LTC_{dyn}-MP)**	MP normalized to dynamic lung-thorax compliance (LTC_{dyn}-MP) was calculated using MP and dynamic lung-thorax compliance (LTC_{dyn})[7]:<br><br>$$LTC_{dyn}\text{-MP } (J/min * cmH_2O/mL) = MP / LTC_{dyn}$$<br>$$LTC_{dyn}\text{-MP } (J/min * cmH_2O/mL) = (0.098 * VT * RR * P_{max}) * (\Delta P_{aw} / VT)$$<br>$$\downarrow$$<br>$$LTC_{dyn}\text{-MP } (cmH_2O^2/min) = RR * P_{max} * \Delta P_{aw}$$<br>$$LTC_{dyn}\text{-MP } (cmH_2O^2/min) = RR * P_{max} * (P_{max} - PEEP)$$<br><br>This formula accounts for different effects of changes in respiratory rate, inspiratory pressure (P_{max}), and PEEP (and thus changes in ΔP_{aw}) on delivered energy. Increasing RR leads to a linear rise in energy transfer while increasing pressure (concomitantly increasing tidal volume) results in an exponential increment in power[3].
**Mechanical power normalized to dynamic lung compliance (C_{lung}-MP)**	Introducing esophageal pressure (P_{es}) to the formula for LTC_{dyn}-MP, thereby replacing P_{max} by absolute end-inspiratory transpulmonary pressure ($P_{L\_end-insp}$) and ΔP_{aw} by the transpulmonary driving pressure (ΔP_L), leads to an equation that estimates stress intensity to the lungs in the pressure-controlled ventilation mode (C_{lung}-MP):<br><br>$$C_{lung}\text{-MP } (cmH_2O^2/min) = RR * P_{L\_end-insp} * \Delta P_L$$<br>$$C_{lung}\text{-MP } (cmH_2O^2/min) = RR * (P_{max} - P_{es\_end-insp}) * ([P_{max} - P_{es\_end-insp}] - [PEEP - P_{es\_end-exp}])$$<br><br>Provided all other variables remain constant, an increase in RR, in absolute $P_{L\_end-insp}$ (i.e., when a decrease in P_{es} occurs) or in ΔP_L (i.e., decreasing PEEP) leads to an increment in MP per ventilated unit of lung volume.
**Power index of the respiratory system (PI_{rs})**	LTC_{dyn}-MP normalized to P_aCO_2 was calculated using LTC_{dyn}-MP and P_aCO_2:<br><br>$$PI_{rs}^X (cmH_2O^2/min) = LTC_{dyn}\text{-MP} * (P_aCO_{2\text{-actual}}/P_aCO_{2\text{-target}})^X$$<br>$$PI_{rs}^X (cmH_2O^2/min) = RR * P_{max} * \Delta P_{aw} * (P_aCO_{2\text{-actual}}/P_aCO_{2\text{-target}})^X$$<br><br>Since P_aCO_2 is inversely proportional to minute ventilation (neglecting dead space fraction), exponent X approximates values between 1 and 2, depending on whether adjustments of ventilator settings are made for RR and/or P_{max} /ΔP_{aw} to reach $P_aCO_{2\text{-target}}$, which was set at 45.0 mmHg (6.0 kPa, corresponding to the hypercapnic threshold) for all patients. PI_{rs} equals the LTC_{dyn}-MP necessary to provide adequate alveolar ventilation (keeping P_aCO_2 below the hypercapnic threshold).

Statistical analysis – Metrics of diagnostic accuracy

ROC curve analysis

To assess the accuracy of the variables analyzed to predict weaning outcome, a receiver operating characteristic (ROC) curve analysis was performed in the entire study population (Fig. S1), and diagnostic performance was expressed as the area under the ROC curve (AUROC). Furthermore, we compared ROC curves of different prediction variables.

k-fold cross-validation

To ensure that the proportion of patients with successful and unsuccessful weaning in both groups reflected the ratio in the whole study population (stratification of outcome), patients were randomly assigned to one of two groups in a stratified, two times repeated, 2-fold cross-validation[8] (groups A/B or C/D) (Fig. S1). Each of the two groups then acted once as a training set and once as a test set. Threshold values that best predicted prolonged weaning failure were derived from the training sets using ROC curve analysis employing the non-parametric method from DeLong[9]. The thresholds that minimized the difference between sensitivity and specificity (assuming equal clinical implications from a false positive and a false negative test) were then used in the test sets to determine the diagnostic performance of each variable. The resulting cross-validated performance of each index, expressed as sensitivity (Sens), specificity (Spec), positive predictive value (PPV), negative predictive value (NPV), accuracy (ACC), positive likelihood ratio (PLR), negative likelihood ratio (NLR), diagnostic odds ratio (DOR)[10], F_1 score, and Matthews correlation coefficient (MCC)[12], equals to the averaged metrics derived from all (four) test sets (Fig. S1, Fig. S2).

Figure S1. Statistical methods – ROC curve analysis, Pearson correlation, logistic regression, and prospective 2-times repeated, 2-fold cross-validation

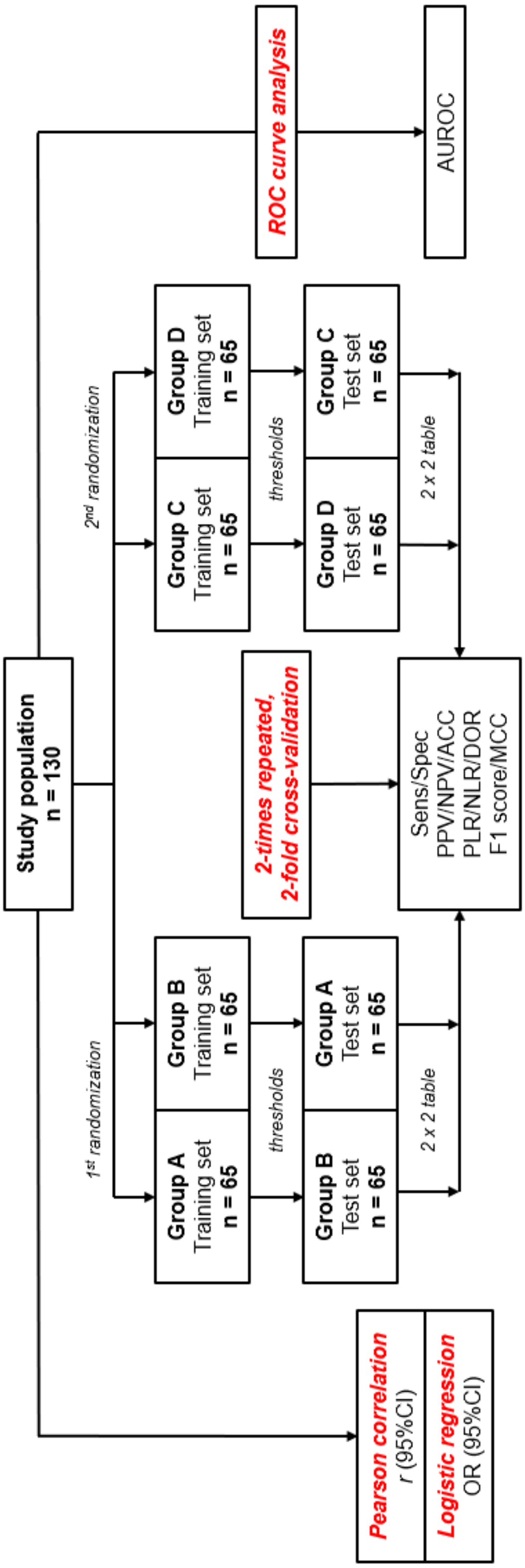

Abbreviations: Sens, sensitivity; Spec, specificity; PPV, positive predictive value; NPV, negative predictive value; ACC, accuracy; PLR, positive likelihood ratio; NLR, negative likelihood ratio; DOR, diagnostic odds ratio; MCC, Matthews correlation coefficient; ROC, receiver operating characteristic (curve); AUROC, area under the ROC curve.

Figure S2. 2 x 2 confusion matrix – Metrics of diagnostic accuracy

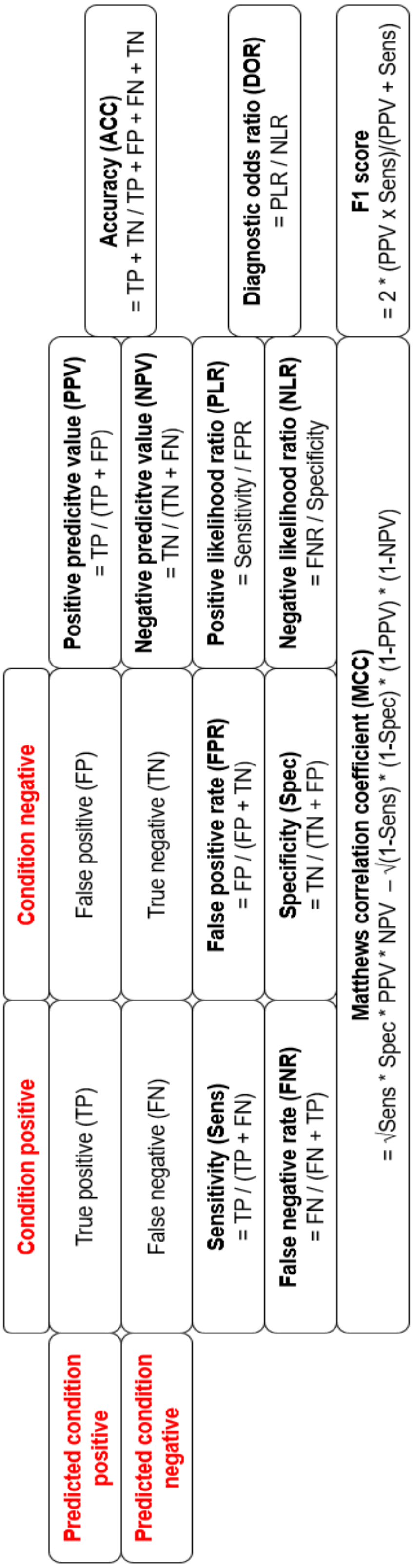

Abbreviations: Sens, sensitivity; Spec, specificity; PPV, positive predictive value; NPV, negative predictive value; ACC, accuracy; PLR, positive likelihood ratio; NLR, negative likelihood ratio; DOR, diagnostic odds ratio; MCC, Matthews` correlation coefficient; ROC, receiver operating characteristic (curve); AUROC, area under the ROC curve.

Table S1. Clinical characteristics on admission to the weaning center – comparison of groups A/B and C/D

**Clinical characteristics**	**Group A (n = 65)**	**Group B (n = 65)**	**Group C (n = 65)**	**Group D (n = 65)**	***P* value**[a]
Age (years)	68 (58–74)	71 (62–76)	67 (59–74)	71 (62–76)	n.s.[b]
Gender (male)	43 (66.2)	39 (60.0)	43 (66.2)	39 (60.0)	n.s.[c]
Body mass index (kg/m^2)	26.4 (23.8–31.1)	26.0 (22.9–31.0)	27.7 (23.6–32.7)	25.4 (22.9–29.0)	n.s.[b]
Obesity (BMI ≥ 30 kg/m^2)	18 (27.7)	22 (33.8)	24 (36.9)	16 (24.6)	n.s.[c]
Smoking history	23 (35.4)	25 (38.5)	24 (36.9)	24 (36.9)	n.s.[c]
APACHE-II (points)	16 (11–19)	16 (13–19)	16 (12–19)	15 (12–19)	n.s.[b]
Albumin (g/dL)	2.1 (1.8–2.5)	2.1 (1.8–2.4)	2.1 (1.9–2.4)	2.1 (1.8–2.5)	n.s.[b]
VD on admission	25 (16–34)	25 (17–35)	26 (16–33)	25 (16–35)	n.s.[b]
ETI to tracheostomy (days)	12 (8–18)	11 (6–17)	12 (7–18)	11 (7–17)	n.s.[b]
ECLA	8 (12.3)	6 (9.2)	9 (13.8)	5 (7.7)	n.s.[c]
**Reason for MV**					
Pneumonia	25 (38.5)	26 (40.0)	27 (41.5)	24 (36.9)	n.s.[c]
Surgery	20 (30.8)	12 (18.5)	12 (18.5)	20 (30.8)	n.s.[c]
Cardiopulmonary resuscitation	5 (7.7)	5 (7.7)	4 (6.2)	6 (9.2)	n.s.[c]
Acute exacerbation of COPD	4 (6.2)	6 (9.2)	4 (6.2)	6 (9.2)	n.s.[c]
Sepsis (including septic shock)	2 (3.1)	5 (7.7)	5 (7.7)	2 (3.1)	n.s.[d]
Acute heart failure	3 (4.6)	3 (4.6)	4 (6.2)	2 (3.1)	n.s.[d]
Other	6 (9.2)	11 (16.9)	10 (15.4)	7 (10.8)	n.s.[c]
**Comorbidities**					
Charlson comorb. index (points)	5 (4–7)	6 (4–7)	6 (4–7)	6 (4–7)	n.s.[b]
Renal insufficiency	21 (32.3)	25 (38.5)	25 (38.5)	21 (32.3)	n.s.[c]
Hemodialysis	11 (16.9)	13 (20.0)	15 (23.1)	9 (13.8)	n.s.[c]
Diabetes mellitus	21 (32.3)	14 (21.5)	20 (30.8)	15 (23.1)	n.s.[c]
Coronary artery disease	16 (24.6)	17 (26.2)	12 (18.5)	21 (32.3)	n.s.[c]
COPD	12 (18.5)	18 (27.7)	14 (21.5)	16 (24.6)	n.s.[c]
Chronic heart failure	5 (7.7)	12 (18.5)	10 (15.4)	7 (10.8)	n.s.[c]
Malignancy	***8 (12.3)***	***2 (3.1)***	6 (9.2)	4 (6.2)	***0.049***[c]
Hepatopathy	3 (4.6)	4 (6.2)	4 (6.2)	3 (4.6)	n.s.[d]
Interstitial lung disease	3 (4.6)	5 (7.7)	4 (6.2)	4 (6.2)	n.s.[d]

Legend

Continuous variables are presented as median (– interquartile range [IQR]); categorical variables are presented as numbers (%).

a: P value for differences between patients in groups A/B and groups C/D; n.s. [not significant] indicates that there was a significant difference neither between groups A and B nor between groups C and D
b: Mann-Whitney *U*-test
c: Chi-squared test
d: Fisher's exact test

Abbreviations: BMI, body mass index; APACHE-II, Acute Physiology and Chronic Health Evaluation II score; VD, ventilator days; ETI, endotracheal intubation; ECLA, extracorporeal lung assistance (in acute respiratory failure); COPD, chronic obstructive pulmonary disease.

Table S2. Reason for failure of first spontaneous breathing trial (n = 30)

Reason for SBT-failure	n (%)
Hypercapnia post-SBT*	20 (15.4)
Acidosis post-SBT*	0 (0.0)
Premature termination of SBT	14 (10.8)
Respiratory rate > 35/min	13 (10.0)
SpO_2 < 88%	11 (8.5)
Heart rate > 130 bpm	1 (0.8)
Systolic blood pressure > 160 mmHg	5 (3.8)

Legend

Categorical variables are presented as numbers (%).

*****: ABG at the end of the SBT was missing in seven patients due to severe respiratory distress, requiring immediate resumption of mechanical ventilation

Abbreviations: SBT, spontaneous breathing trial; bpm, beats per minute.

Table S3. Thresholds of variables used to predict the outcome of first SBT – mean values derived from the training sets

Variables	Thresholds
P_aCO_2 on MV (mmHg)	> 35.8
Ventilatory ratio	> 1.22
LTC_{dyn} (mL/cmH_2O)	≤ 28.4
Mechanical power (J/min)	> 20.9
PBW-MP (J/min/kg)	> 0.3344
LTC_{dyn}-MP (cmH_2O^2/min)	> 7766
Power index$_{rs}^{1.0}$ (cmH_2O^2/min)	> 6083
Power index$_{rs}^{2.0}$ (cmH_2O^2/min)	> 4779

Legend

> / ≤ indicate whether values above/below the threshold value predicted failure of the SBT. The associated criterion is the threshold value that minimized the difference between sensitivity and specificity of the test, graphically corresponding to the intersection of the line connecting the left-upper corner and the right-lower corner of the unit square and the ROC curve.

Abbreviations: SBT, spontaneous breathing trial; MV, mechanical ventilation; LTC_{dyn}, dynamic lung-thorax compliance; PBW-MP, mechanical power normalized to predicted body weight; LTC_{dyn}-MP, mechanical power normalized to dynamic lung-thorax compliance.

Table S4: Multivariable model 1 – Results of binary logistic regression analysis[1]

**Logit(p)**	L = – 9.413 + (0.084 * MP) + (0.176 * P_aCO_2 on MV)		
**OR (95%CI); *P* value**	**MP**	1.09 (1.00–1.18)	0.043
	**P_aCO_2 on MV**	1.19 (1.09–1.31)	< 0.01
**Hosmer & Lemeshow**	0.669		
**Nagelkerke R^2**	0.259		
**AUROC (95%CI)**	0.76 (0.69 – 0.84)		
**2 x 2 table**	**TP** 11		**FP** 5
	**FN** 19		**TN** 95
**Metrics from 2 x 2 table (95%CI)**	**Sensitivity**	37% (20–56)	**PLR** 7.3
	**Specificity**	95% (89–98)	**NLR** 0.7
	**PPV**	69% (45–85)	**DOR** 11
	**NPV**	83% (79–87)	**F_1 score** 0.48
	**Accuracy**	82% (74–88)	**MCC** 0.41

Legend

Abbreviations: L, log-odds/Logit(p); MP, mechanical power; MV, mechanical ventilation; OR, odds ratio; 95%CI, 95% confidence interval; AUROC, area under the receiver operating characteristic curve; TP, true positive; FP, false positive; FN, false negative; TN, true positive; PPV, positive predictive value; NPV, negative predictive value; PLR, positive likelihood ratio; NLR, negative likelihood ratio; DOR, diagnostic odds ratio; MCC, Matthews correlations coefficient[12]

Table S5: Multivariable model 2 – Results of binary logistic regression analysis[1]

**Logit(p)**	L = – 11.609 + (0.120 * PBW-MP * 10^2) + (0.260 * P_aCO_2 on MV) + (- 2.564 * VR)		
**OR (95%CI); *P* value**	**PBW-MP * 10^2**	1.13 (1.05–1.21)	< 0.01
	**P_aCO_2 on MV**	1.30 (1.14–1.47)	< 0.01
	**VR**	0.08 (0.01–0.92)	0.043
**Hosmer & Lemeshow**	0.749		
**Nagelkerke R^2**	0.340		
**AUROC (95%CI)**	0.82 (0.74 – 0.88)		
**2 x 2 table**	**TP** 11		**FP** 6
	**FN** 19		**TN** 94
**Metrics from 2 x 2 table (95%CI)**	**Sensitivity**	37% (20–56)	**PLR** 6.1
	**Specificity**	94% (87–98)	**NLR** 0.7
	**PPV**	65% (43–82)	**DOR** 9
	**NPV**	83% (79–87)	**F_1 score** 0.47
	**Accuracy**	81% (73–87)	**MCC** 0.39

Legend

Abbreviations: L, log-odds/Logit(p); PBW-MP, mechanical power normalized to predicted body weight; MV, mechanical ventilation; VR, ventilatory ratio; OR, odds ratio; 95%CI, 95% confidence interval; AUROC, area under the receiver operating characteristic curve; TP, true positive; FP, false positive; FN, false negative; TN, true positive; PPV, positive predictive value; NPV, negative predictive value; PLR, positive likelihood ratio; NLR, negative likelihood ratio; DOR, diagnostic odds ratio; MCC, Matthews correlations coefficient[12]

Table S6: Multivariable model 3 – Results of binary logistic regression analysis[1]

**Logit(p)**	L = – 8.520 + (0.221 * LTC_{dyn}-MP * 10^{-3}) + (0.154 * P_aCO_2 on MV)			
**OR (95%CI); *P* value**	**LTC_{dyn}-MP * 10^{-3}**	1.25 (1.06–1.46)		< 0.01
	**P_aCO_2 on MV**	1.17 (1.06–1.28)		< 0.01
**Hosmer & Lemeshow**	0.960			
**Nagelkerke R^2**	0.293			
**AUROC (95%CI)**	0.80 (0.72 – 0.87)			
**2 x 2 table**	**TP** 11		**FP** 5	
	**FN** 19		**TN** 95	
**Metrics from 2 x 2 table (95%CI)**	**Sensitivity**	37% (20–56)	**PLR**	7.3
	**Specificity**	95% (89–98)	**NLR**	0.7
	**PPV**	69% (45–85)	**DOR**	11
	**NPV**	83% (79–87)	**F_1 score**	0.48
	**Accuracy**	82% (74–88)	**MCC**	0.41

Legend

Abbreviations: L, log-odds/Logit(p); LTC_{dyn}-MP, mechanical power normalized to dynamic lung-thorax compliance; MV, mechanical ventilation; OR, odds ratio; 95%CI, 95% confidence interval; AUROC, area under the receiver operating characteristic curve; TP, true positive; FP, false positive; FN, false negative; TN, true positive; PPV, positive predictive value; NPV, negative predictive value; PLR, positive likelihood ratio; NLR, negative likelihood ratio; DOR, diagnostic odds ratio; MCC, Matthews correlations coefficient[12]

Table S7. Pearson correlation between prediction variables and duration of first SBT – all patients

**Variables**	**Pearson correlation coefficient (*r*)**	**P value**
P_aCO_2 on MV	- 0.26 (-0.42 – -0.10)	**0.002**
Ventilatory ratio	- 0.24 (-0.39 – -0.07)	**0.006**
LTC_{dyn}	0.15 (-0.03 – 0.31)	**0.010**
Mechanical power	- 0.25 (-0.40 – -0.08)	**0.005**
PBW-MP	- 0.22 (-0.38 – -0.05)	**0.011**
LTC_{dyn}-MP	- 0.28 (-0.43 – -0.11)	**0.001**
Power $index_{rs}$ $^{1.0}$	- 0.35 (-0.49 – -0.19)	**< 0.001**
Power $index_{rs}$ $^{2.0}$	- 0.38 (-0.52 – -0.22)	**< 0.001**

Legend

The correlation of each variable in the whole study population with the duration of the first SBT presented as Pearson's *r* (with 95% confidence intervals).

Abbreviations: SBT, spontaneous breathing trial; MV, mechanical ventilation; LTC_{dyn}, dynamic lung-thorax compliance; PBW-MP, mechanical power normalized to predicted body weight; LTC_{dyn}-MP, mechanical power normalized to dynamic lung-thorax compliance.

Figure S3. Scatter diagram – Correlation of the Power index$_{rs}^{2.0}$ with P_aCO_2 at the end of SBT

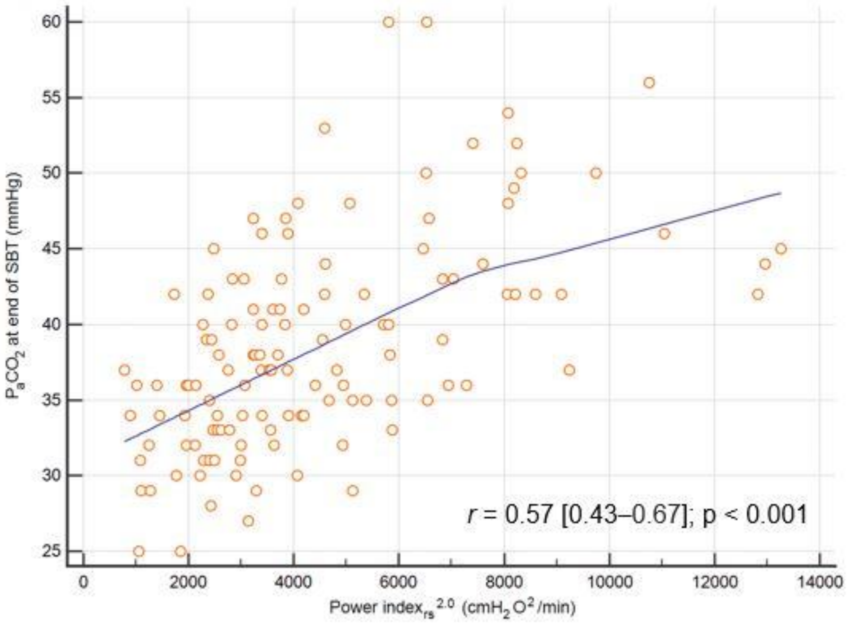

Legend
The correlation of the Power index$_{rs}^{2.0}$ in the whole study population with P_aCO_2 at the end of SBT

Abbreviations: SBT, spontaneous breathing trial; *r*, Pearson's *r* (95% confidence intervals).

References

1. Sinha, P., et al. Ventilatory ratio: a simple bedside measure of ventilation. *Br J Anaesth*. **102**, 692–97 (2009)
2. Sinha, P., et al. Analysis of ventilatory ratio as a novel method to monitor ventilatory adequacy at the bedside. *Crit Care*. **17**, R34 (2013)
3. Gattinoni, L., et al. Ventilator-related causes of lung injury: the mechanical power. *Intensive Care Med*. **42**, 1567–1575 (2016)
4. Becher, T., et al. Calculation of mechanical power for pressure-controlled ventilation. *Intensive Care Med*. **45**, 1321–23 (2019)
5. Chiumello, D., et al. Bedside calculation of mechanical power during volume- and pressure-controlled mechanical ventilation. *Crit Care*. **24**, 417 (2020)
6. Brower, R.G., et al. Ventilation with lower tidal volumes as compared with traditional tidal volumes for acute lung injury and the acute respiratory distress syndrome. *N Engl J Med*. **342**, 1301–1308 (2000)
7. Ghiani, A., et al. Variables predicting weaning outcome in prolonged mechanically ventilated tracheotomized patients: a retrospective study. *J Intensive Care.* **8**, 19 (2020)
8. Arlot, S., et al. A survey of cross-validation procedures for model selection. *Stat Surv*. **4**, 40–79 (2010)
9. DeLong, E.R., et al. Comparing the areas under two or more correlated receiver operating characteristic curves: a non-parametric approach. *Biometrics*. **44**, 837–845 (1988)
10. Fischer, J.E., et al. A readers` guideline to the interpretation of diagnostic test performance: clinical example of sepsis. *Intensive Care Med*. **29**, 1043–1051 (2003)
11. Leisman DE, et al. Development and reporting of prediction models: Guidance for authors from editors of respiratory, sleep, and critical care journals. *Crit Care Med.* **48**, 623–633 (2020)
12. Chicco, D., et al. The advantages of the Matthews correlation coefficient (MCC) over F1 score and accuracy in binary classification evaluation. *BMC Genomics.* **21**, 6 (2020)
